# The immune response in canine and human leishmaniasis and how this influences the diagnosis- a review and assessment of recent research

**DOI:** 10.3389/fcimb.2023.1326521

**Published:** 2023-12-08

**Authors:** Larisa Ivănescu, Bianca Lavinia Andronic, Smaranda Grigore-Hristodorescu, Gabriela Victoria Martinescu, Raluca Mîndru, Liviu Miron

**Affiliations:** ^1^ Clinics Department, Faculty of Veterinary Medicine, Iasi University of Life Sciences, Iaşi, Romania; ^2^ Epidemiology Department, Faculty of Medicine, Grigore T. Popa University of Medicine and Pharmacy, Iaşi, Romania

**Keywords:** leishmaniasis, immunological status, diagnostic techniques, HIV co-infection, serological, molecular, parasitological

## Abstract

Leishmaniasis is a widespread but still underdiagnosed parasitic disease that affects both humans and animals. There are at least 20 pathogenic species of *Leishmania*, most of them being zoonotic. The diagnosis of leishmaniasis remains a major challenge, with an important role being played by the species of parasites involved, the genetic background, the immunocompetence of the host. This paper brings to the fore the sensitivity of the balance in canine and human leishmaniasis and addresses the importance of the host’s immune response in establishing a correct diagnosis, especially in certain cases of asymptomatic leishmaniasis, or in the situation the host is immunosuppressed or acquired leishmaniasis through vertical transmission. The methods considered as a reference in the diagnosis of leishmaniasis no longer present certainty, the diagnosis being influenced mostly by the immune response of the host, which differs according to the presence of other associated diseases or even according to the breed in dogs. Consequently, the diagnosis and surveillance of leishmaniasis cases remains an open topic, requiring new diagnostic methods adapted to the immunological state of the host.

## 1 Introduction

Leishmaniasis is a vector-borne, parasitic disease produced by more than 20 species of protozoa belonging to the genus *Leishmania*. The vectors are represented by approximately 90 species of sandflies (World Health Organization. Leishmaniasis, 2023). The parasite is either identified as a promastigote, which develops in the vector or as an amastigote, which develops in the host targeting the reticuloendothelial system in a multitude of tissues, mainly occurring in the bone marrow, the lymph nodes, the spleen and the liver (Van Griensven and Diro, 2012; Van Griensven and Diro, 2019).

Leishmaniasis can have three forms: 1. visceral leishmaniasis (VL), usually caused by *L. donovani* and *L. infantum* (also known as *L. chagasi*) (CDC, 2023), referred to also as kala-azar, with approximately 50,000 to 90,000 new cases of leishmaniasis occurring annually globally, but with only 25-45% of them being reported to the WHO (World Health Organization. Leishmaniasis, 2023); 2. cutaneous leishmaniasis (CL), caused by *Leishmania tropica, L. major, L. aethiopica, L. infantum*, *L. donovani* in the Old World and in the New World by species from *L. mexicana* complex or from *Viannia* subgenus, and also *L. infantum/L.chagasi* (CDC, 2023). This form is considered to be the most frequent form (approximately 95% of cases taking place in the Americas, the Middle East, the Mediterranean basin and Central Asia), with an annual estimate from 600,000 to 1 million new cases throughout, which are unfortunately underreported, as only approximately 200,000 are reported to the WHO (Reithinger et al., 2007; Alvar et al., 2012; World Health Organization. Leishmaniasis, 2023); 3. mucocutaneous leishmaniasis (caused *by L. amazonensis* and by species of the *Viannia* subgenus) (CDC, 2023) with over 90% of cases reported in Bolivia (plurinational state), Ethiopia, Brazil and Peru. Manifestations of the disease are different depending on the species of parasite and on the state of the host’s immune system, ranging from fatal to self-limiting (Torres-Guerrero et al., 2017). Immune control can be achieved through the activation of macrophages and an intact T-helper cell type 1 re-sponse (Th1); which underlines the major risk from visceral leishmaniasis, in case of immunosuppression (Van Griensven et al., 2014).

The dog represents the main domestic reservoir for *Leishmania infantum* (Baneth et al., 2008; Travi et al., 2018; Marcondes and Day, 2019). Bihar State in India comprises over 90% of visceral leishmaniasis cases, making it the fourth most common tropical disease in morbidity and second only to malaria in mortality (Pace, 2014). In the Mediterranean basin, China, the Middle East and South America the zoonotic form appears, produced by *L. infantum*, with the main reservoir being the dog (Van Griensven and Diro, 2012). In East Africa, Nepal, Bangladesh and India, the anthroponotic form, produced by *L. donovani*, is predominantly found (Van Griensven and Diro, 2019). A study carried out in Brazil, using the PCR technique, demonstrated a prevalence of asymptomatic leishmaniasis infection of up to 80% in the analysed dogs (Courtenay et al., 2002; Pederiva et al., 2023). The processes of asymptomatic infection and the body’s immunological reactions are not fully understood, which is why diagnostic methods must be adapted for each individual patient (Burza et al., 2018; Mody et al., 2019).

Domestic and wild canids are considered the main parasite reservoir, maintaining a continuous cycle of transmission of *L. chagasi* in the New World and of *L. infantum* in the Old World. In Europe, a particular interest (from the public health point of view and from the veterinary pharmaceutical field) is shown for canine visceral leishmaniasis (CVL), a serious disease manifested through a chronic evolution of viscerocutaneous signs. It is considered that no less than 2.5 million dogs manifest this form of disease, developing severe forms with a high degree of death (Athanasiou et al., 2012). Both symptomatic and asymptomatic dogs can be regarded as a natural reservoir of *L. infantum* for both dogs and humans (Otranto and Dantas-Torres, 2013), being a real danger in the endemic regions of Latin America and the Mediterranean (Grimaldi et al., 2017).

## 2 The immune response in human leishmaniasis

In most individuals, leishmaniasis does not progress to a noticeable form of the disease. In regions with a high degree of endemicity, up to 30% of infected residents are asymptomatic (Michel et al., 2011). Control of infection is based on activated, leishmanicidal macrophages and an intact specific Th1 cell response. Cell-mediated immunity is indicated through a positive skin test result. The observable disease form is associated with a mixed Th1/T-helper type 2 (Th2) response. Increased levels of regulatory T cells contribute to the severe immunosuppression observed in visceral leishmaniasis (Murray et al., 2005). The Th1 cellular immune response is characterized by the production of proinflammatory cytokines like interferon- γ (IFN γ) and Tumor Necrosis Factor alpha (TNF α) (Kushwaha and Kaur, 2021). The Th2 immune response is characterized by the production of IL-10 and Transforming Growth Factor β (TGFβ), which supports disease progression (Costa-da-Silva et al., 2022). In case of immunosuppression, a reactivation of the disease can occur, as the parasites remain viable after the primary infection (Bogdan, 2008; Van Griensven and Diro, 2019). In HIV infection, a depletion of CD4 T cells is observed, with a bias toward a Th2-type immune response, thus affecting both the innate and adaptive immune systems, along with macrophage functionality (Ortiz and Silvestri, 2009). HIV and *Leishmania* spp. both invade cells belonging to the monocyte lineage (macrophage, dendritic cells), so a co-infection with HIV increases the pathogenicity of the disease (Andreani et al., 2012).

In most cases of HIV co-infections recorded in Europe, a CD4 cell count of <200 cells/ml was recorded (Ortiz and Silvestri, 2009); but studies in Ethiopia (where *L. donovani* is predominant) showed higher CD4 cell counts (Santos-Oliveira et al., 2010). The immunosuppression which favours visceral leishmaniasis, creating a predisposition to it, can also be induced by a series of immunosuppressive or immunomodulatory treatments, significantly affecting T-cell lymphocytes (Chinen and Buckley, 2010). Visceral leishmaniasis increases the rate of onset of AIDS and decreases the lifetime of HIV infected patients (Pintado et al., 2001). It should be taken into account that in humans co-infected with the HIV virus, the parasites can be found in abnormal tissues like the intestine, the oral cavity, lung tissue or skin (Puig and Pradinaud, 2003; Jeziorski et al., 2009; Van Griensven et al., 2014). Post-kala azar dermal leishmaniasis was reported in higher numbers in immunosuppressed patients (Ritmeijer et al., 2001). This dermal form in *L. infantum* infection is not usually observed but it was reported in HIV patients (Rihl et al., 2006; Stark et al., 2006).

In visceral leishmaniasis there is an overactivation of B cells, which leads to a marked polyclonal hypergammaglobulinemia, which may result in a positive indirect Coombs test and detectable levels of antinuclear antibodies, anti-cardiolipin antibodies, anti-dsDNA antibodies and rheumatoid factor IgM (Sakkas et al., 2008). Circulating immune complexes, cryoglobulinemia and low complement levels could also be noticed (Atta et al., 2007), similar symptomatology being able to lead to a misdiagnosis of an autoimmune disease like rheumatoid arthritis or systemic lupus erythematosus and/or as an onset of the basic disease (Voulgari, 2004; Venizelos et al., 2009).

In *Leishmania* spp. infection, innate immune cells can modulate their function and phenotype, as well as a series of adaptive immune responses. So when we talk about a progression of the disease, mast cells secrete IL-4 and IL-13, which favour Th2 responses and at the same time the survival of the parasites. Macrophages, neutrophils and dendritic cells (DC) can eliminate parasites or promote their survival. The elimination of leishmanial parasites by the recruited neutrophils is done by releasing the phagocytosis process, ROS (reactive oxygen species) and NET (neutrophil extracellular traps), IL-8 and MIP-1β (macrophage inflammatory protein – 1 beta) are secreted, which attract additional neutrophils. By inhibiting phagolysosome biogenesis and oxidative stress and delaying neutrophil apoptosis, the *Leishmania* parasite can transiently survive.

M1 macrophages are responsible for the production of pro-inflammatory cytokines and chemokines, leading to the stimulation of Th1 responses, thus favouring disease control. M2 macrophages lead to increased production of IL-10 and TGFβ resulting in Th2 response and thus disease progression. IL-10 has been shown to be able to inhibit phagocytosis, thus contributing to the growth and spread of the Leishmania parasite (Kane and Mosser, 2001; Gautam et al., 2011); it acting as an immunosuppressive factor in VL and facilitates the spread of parasites. Secretion of IL-10 by T cells can influence immune activation early in infection, leading to susceptibility to uncontrolled major infection (Schwarz et al., 2013).

**Figure 1:**
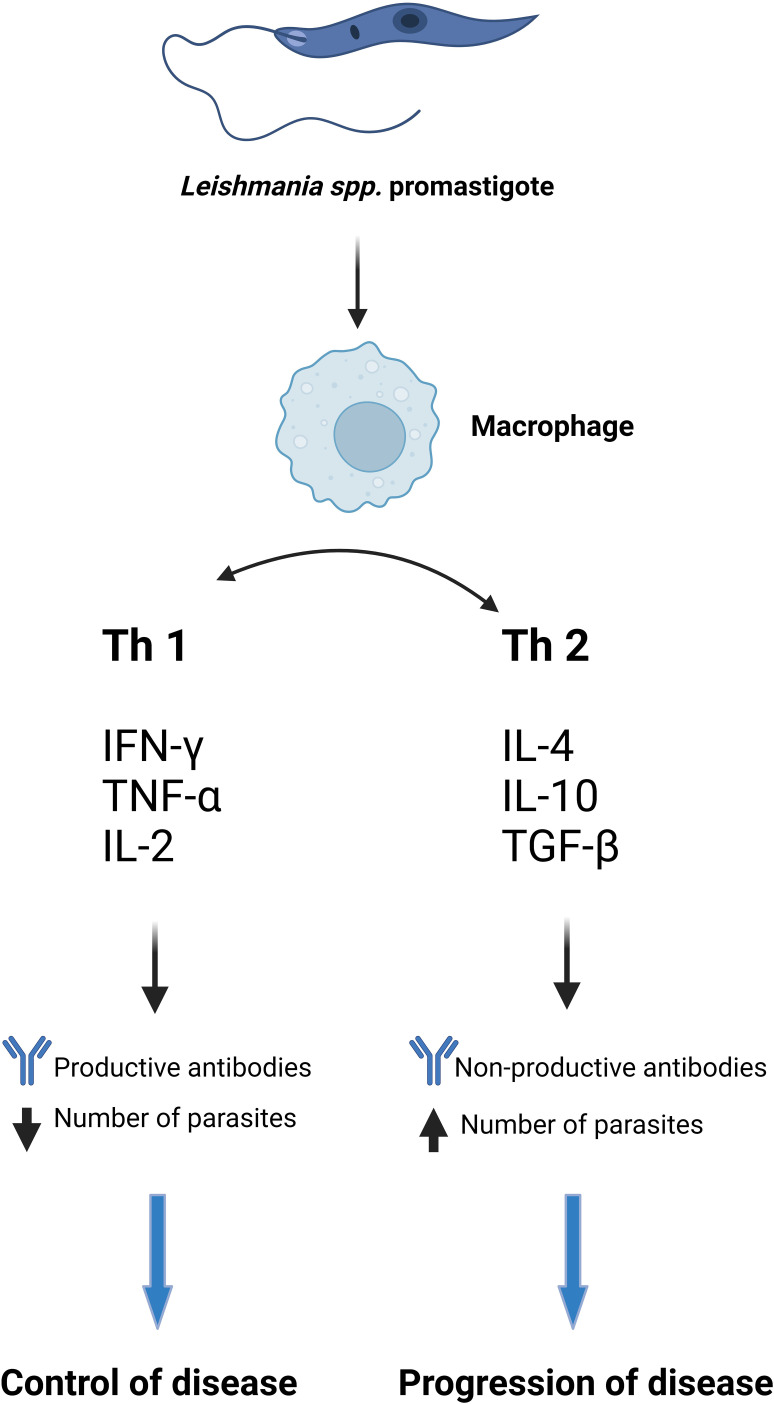
The immune response in CVL and VL (Gantt et al., 2001; Kane and Mosser, 2001; Mukbel et al., 2007; Ehrchen et al., 2010; Filardy et al., 2010; Gautam et al., 2011; Deschacht et al., 2012; Sena and Chandel, 2012; Arango Duque and Descoteaux, 2015; Rochael et al., 2015; Carneiro et al., 2016; Charmoy et al., 2016; Loría-Cervera and Andrade-Narvaez, 2020). Created with BioRender.com.

Thus DCs can induce Th1 differentiation by secreting IL-12 and IL-27 or Th2 by blocking IL-12 secretion. NK (natural killer) cells secrete IFNγ stimulating the Th1 response and thus having a protective role in leishmaniasis (Gantt et al., 2001; Mukbel et al., 2007; Ehrchen et al., 2010; Filardy et al., 2010; Deschacht et al., 2012; Sena and Chandel, 2012; Arango Duque and Descoteaux, 2015; Rochael et al., 2015; Carneiro et al., 2016; Charmoy et al., 2016; Loría-Cervera and Andrade-Narvaez, 2020). It has been proven that interleukin-12 or IFN treatment offers the possibility of modulating the rapid response to IL-4, which plays a very important role in the maturation of Th1 and Th2 cells (Aseffa et al., 2002). Interleukin-12 (IL-12) is a key cytokine in driving the immune system towards a Th1 response and preventing the Th2 immune profile. Therefore, IL-12 is indispensable in the defense against certain pathogens, mainly intracellular, but the overproduction of this cytokine is crucially involved in the etiology of several inflammatory and autoimmune diseases (Haskó and Szabó, 1999). The Th1 immune response plays an extremely important role in protecting the host against primary infection, but also provides lifelong immunity to reinfection (McMahon-Pratt and Alexander, 2004; Amit et al., 2017) (**Figure 1**).

## 3 Diagnosis in human leishmaniasis

Firstly, after the sandfly bite, a small erythema appears, followed by an inflammatory reaction caused by the parasite, which can develop into an open ulcer or infiltrate in organs like liver or spleen (Reithinger and Dujardin, 2007). Great importance must be given to the early diagnosis of leishmaniasis, since this can prevent both the evolution of severe clinical signs and mortality in humans with visceral leishmaniasis.

The diagnosis of leishmaniasis is challenging, based on history, clinical evaluation and laboratory data (Thakur et al., 2020; Santos et al., 2021). The parasitological diagnosis of leishmaniasis represents the gold standard, and it consists in the detection of parasites in tissue fragments or from organ aspirates (Elmahallawy et al., 2014; Shirian et al., 2014), or direct histology/microscopy in the case of cutaneous leishmaniasis (Srivastava et al., 2011). However, this method is less and less used, as sample collection is invasive and requires skilled personnel trained in both protozoan identification and collection technique (Barrett and Croft, 2012; Srividya et al., 2012; Pessoa-e-Silva et al., 2019). Thus, the lack of diagnostic certainty greatly limits disease control and contributes to the maintenance of a reservoir in nature. Immunological methods are most commonly used because they are not invasive, although their sensitivity and/or specificity have presented variability (Mettler et al., 2005; Pattabhi et al., 2010; Das et al., 2011; Reimão et al., 2020). Recently, leishmaniasis diagnosis has been based on the evaluation of specific proteins (Faria et al., 2015; Farahmand and Nahrevanian, 2016). In symptomatic visceral leishmaniasis, there is an increased antibody response, both in dogs and in humans, and recombinant antigen-based assays can thus be used (Dhom-Lemos et al., 2019; Lévêque et al., 2020; Pereira et al., 2020). The challenge is asymptomatic leishmaniasis, in which we face a low serology against the protozoan, the tests showing a reduced sensitivity, as well as a cross-reactivity with other pathologies such as tuberculosis, Chagas disease, or malaria, which reduces the tests’ specificity (Daltro et al., 2019; Rodrigues et al., 2019). Recently, the rLiHyQ protein was identified in the antigens of *Leishmania* spp.; this was cloned and used in ELISA experiments for determining the diagnosis in both human and canine leishmaniasis, showing sensitivity and specificity even in HIV-coinfected patients (Santos et al., 2021). A study on an rK39 protein identified in a strain of *L. infantum* from Iran reported a sensitivity of 100% in humans and 97.6% in dogs in the ELISA test, as well as very good results in cross-assays, suggesting that it may represent a reliable diagnosis of canine and human visceral leishmaniasis in Iran (Hosseini Farash et al., 2020). The host’s immune status presents great importance in leishmaniasis diagnosis, for this reason the protocol differs in HIV patients. In 1985, the first report of leishmaniasis associated with HIV infection appeared, with the number subsequently increasing in southern Europe and 35 other countries reporting cases of co-infection. Their number decreased after antiretroviral therapy was introduced (Alvar et al., 2008).

### 3.1 Parasitological methods of diagnosis

This technique still stands as the gold standard in leishmaniasis diagnosis, showing different sensitivity depending on the organ addressed (**Table 1**). In HIV-infected patients, microscopy of splenic aspirate shows the highest sensitivity; the one from the bone marrow presents a sensitivity of 81%, but with the specification that many cases came out positive after repeating the examination of the bone marrow preparation (Besada et al., 2013). Culture has the advantage that it can increase the sensitivity more and also allows species identification, but has the disadvantage that it can take several weeks, that is why microculture is recommended (Ates et al., 2013). Although it is a very specific technique, the sensitivity given by the microscopic detection of parasites is influenced by the smear quality, the experience of the specialist and the reagents used.

**Table 1:** The sensitivity and specificity rates acquired through the parasitological examination.

Direct parasitological examination (microscopy/culture)	Sensitivity(%)	Specificity(%)	References
Aspirated from the spleen (more common in East Africa and Indian subcontinent)	93%-99%; 81% in HIV patients;	>95% in patients with HIV coinfection;	(Alvar et al., 2008; Srivastava et al., 2011; Barrett and Croft, 2012)
Bone marrow aspiration/biopsy (more common in Europe, Brazil, and the United States)	53%–86%	67-94% in patients with HIV coinfection and up to 100% in other cases;	(Van Griensven and Diro, 2012; Elmahallawy et al., 2014; Van Griensven et al., 2014)
Lymph node aspiration	53%–65%	>95%	(Boelaert et al., 2007; Shirian et al., 2014; Guerra et al., 2019)
Peripheral blood from mononuclear cells (PBMCs)	52%	91%	(Hide et al., 2007; Maurya et al., 2010; Galdino et al., 2016)

### 3.2 Serological testing

For the detection of anti-leishmanial antibodies, serological tests include enzyme-linked immunosorbent assay (ELISA), direct agglutination test (DAT), lateral flow immunochromatographic test (ICT) which uses the recombinant antigen rK39 and the immunofluorescence antibody test (IFAT) (Singh et al., 1995; Schallig et al., 2001).

In serological testing, the immunological status of the patients should be considered and in the immunocompetent patients, indirect tests with fluorescent antibodies are very useful in the diagnosis of visceral leishmaniasis, particularly in paediatric cases (Cruz et al., 2006). The most frequently used tests are the direct agglutination test and the rK39 rapid test, although the latter in Sri Lanka did not give the expected results, suggesting that local parasite antigenic variation may also have an influence (Siriwardana et al., 2017). In Brazil, India and Nepal, the ICT rK39 test has become the standard in the diagnosis of leishmaniasis (Bezuneh et al., 2014). The use of rK28 antigens in an RDT-type format gave superior results to rK39, with a sensitivity of 95.9% and specificity of 100% (Pattabhi et al., 2010). It has also been tested in patients with HIV co-infection, in whom serological test results are known to be poorer, but with a very good response between 92.3% and 100% (Bezuneh et al., 2014).

The ELISA test showed satisfactory results in the diagnosis of symptomatic visceral leishmaniasis, being less expensive in comparison to *in-vitro* cultures and PCR tests from bone marrow or other elective organs, but in immunocompromised individuals showed poor values (Bezuneh et al., 2014; Deepachandi et al., 2023).

The DAT presents a very good sensitivity, but it has a number of drawbacks, as it requests a laboratory equipped with a temperature-controlled incubator, overnight incubation, skilled personnel, which makes this test inoperative at the point of care, and also, even if it can detect low levels of antibodies, because it uses multiple antigens it affects the specificity (Gidwani et al., 2011; Ostyn et al., 2011).

An up-to-date commercial test is now available for the detection of antigens, namely the latex agglutination test (KATEX) which is used for leishmanial antigen detection in the urine of patients which present visceral leishmaniasis (Attar et al., 2001). Although the sensitivity is not very good (60–80%), this antigen-based test can represent a real success in visceral leishmaniasis diagnosis in immunocompromised individuals who may have low antibody titres (Riera et al., 2004; Hatam et al., 2009). HIV/AIDS patients receiving antiretroviral treatment develop an asymptomatic leishmaniasis, with an increase in the parasite load, increasing also the risk of infection of sandfly vectors and therefore of disease transmission (Das et al., 2011; Molina et al., 2020).

The use of serological tests is therefore less reliable in patients with HIV co-infection. In Europe it has been shown that more than 40% of these serological tests can give negative results in those patients (Medrano et al., 1998; Van Griensven et al., 2014). However, studies have shown that serological test results are different in HIV patients, as opposed to other categories of immunocompromised patients like those who received organ transplants, in whom for example, the direct agglutination test had a high sensitivity of 80% in East Africa (Cota et al., 2012). Also, the use of the indirect fluorescent antibody test showed a sensitivity of 48% in HIV patients and 93% in transplant patients (Antinori et al., 2008). A summary of the sensitivity and specificity of different serological tests used in leishmaniasis diagnosis is showed in **Table 2**.

**Table 2:** The sensitivity and specificity rates acquired through the serological tests.

	Sensitivity (%)	Specificity (%)	References
Recombinant antigen rK39 (India and Nepal)	> 95.8	100	(Bern et al., 2000; Maia et al., 2012; Bezuneh et al., 2014; Gao et al., 2015; Deepachandi et al., 2023)
Direct agglutination test (DAT)	91.6 (84.1–96.3)	97.3 (92.3–99.4)	(Mikaeili et al., 2007; Bezuneh et al., 2014)
Recombinant antigen rK28	95.9	100	(Pattabhi et al., 2010)
The immunofluorescence antibody test (IFAT)	87.5	95.8	(Alam et al., 1996; Mikaeili et al., 2007; Mniouil et al., 2018)
Immunosorbent assay (ELISA)	75	95,8	(Alam et al., 1996; Mniouil et al., 2018)

In conclusion, the overall performance of rapid rk39 test had a score higher than ELISA and IFAT with a sensitivity and specificity of (95.8%) and (100%) respectively. This immunochromatographic test is non-invasive, rapid, easy to perform and low-cost, recommended to be used for the diagnosis of leishmaniasis, but a second method of diagnostic is still mandatory to have a greater certainty of the result obtained, especially if we are talking about patients with co-infections, especially HIV, because both diseases target the same immune cells (Pintado et al., 2001).

### 3.3 Molecular techniques

The PCR techniques are recommended for samples that have a lower parasite load (Antinori et al., 2007). Moreira et al. (2007) (Moreira et al., 2007) carried out a comparative study of different diagnostic methods, observing a sensitivity of 100%, 96% and 95.65% for symptomatic, oligosymptomatic and asymptomatic patients respectively and 100% specificity.

Taken strictly as a diagnostic method, the PCR method has the disadvantage that it cannot differentiate between active visceral leishmaniasis and asymptomatic infections (Sudarshan and Sundar, 2014), which present a high risk of contamination, these disadvantages can be overcome by quantitative PCR (qPCR), being able to evaluate the levels of infection in the healthy endemic population (Sudarshan et al., 2011; Srivastava et al., 2013).

#### 3.3.1 Multiplex PCR

In this method, different DNA targets can be simultaneously amplified for the diagnosis of leishmaniasis using different types of markers, such as multicopy SL RNAs and minicircle kDNA, but it shows a lower sensitivity compared to the single PCR method (Thakur et al., 2020).

#### 3.3.2 Real-time PCR (quantitative PCR)

This method helps to monitor the parasite load in different tissues throughout the course of the disease and after treatment (Manna et al., 2008). It presents the disadvantage that it is expensive and requires qualified personnel for interpretation. Using peripheral blood for the diagnosis of visceral leishmaniasis, a sensitivity and specificity of 91.3% and 29.6% respectively for Real-Time PCR and 97.78% and 61.82% for classic PCR have been demonstrated (Da Costa Lima et al., 2013). But the sensitivity for the classic PCR technique is dependent on the concentration of DNA in the sample. Thus, emphasis was placed on the Real-Time PCR technique, succeeding in the identification of parasites without cross-amplification with another parasite subgenus (Colombo et al., 2015).

## 4 Immunological response of visceral leishmaniasis in dogs

Visceral leishmaniasis in dogs is very variable, being able to evolve from very severe symptomatic forms to asymptomatic or mild forms classified as oligosymptomatic. The clinical picture includes anaemia, lymphadenopathy, alopecia, diarrhoea, weight loss, onychogryphosis, locomotor problems, epistaxis, conjunctivitis, muscle atrophy, polyuria, polydipsia (Ciaramella et al., 1997; Baneth et al., 2008; Chagas et al., 2021), the range of clinical symptoms being connected to the genetic makeup and immune response of individual animals, which can have a direct impact on their vulnerability or resistance to infection (Baneth, 2006). A big problem for public health, especially in Brazil, is characterized by dogs without clinical manifestations, because they represent a reservoir in nature, being an infection source for vectors (Laurenti et al., 2013; Silva et al., 2016). The non-specific symptomatology is related to the heterogeneity of immune responses, which occurs in leishmaniasis (Hosein et al., 2017). Thus, in leishmaniasis there can be a severe form, with the suppression of the cellular response and the formation of a high titre of antibodies, or there can be an asymptomatic form with the mounting of a protective immune response which leads to a positive or negative serology (Grimaldi et al., 2017). As a mandatory intracellular parasite with localisation in cells of the myeloid system (macrophages, monocytes, neutrophils, antigen-presenting cells), the protozoan has a distinctive and intricate effect on the immune system. In canine leishmaniasis, the first to interact with parasites are neutrophils, which can lead to parasite destruction by an oxidative burst or prolonged parasite survival (Almeida et al., 2013). During disease with clinical manifestations, *Leishmania* protozoa are thought to induce early apoptosis of neutrophils. Parasites manipulate the macrophage immune response within the parasitophorous vacuole by keeping neutrophils alive for an extended period. This enables the parasites to survive in the parasitophorous vacuole and be absorbed by macrophages without causing inflammatory reactions, ultimately resulting in parasite persistence (Regli et al., 2017).

In CVL, immunological changes involving T cells are taking part in, such as the absence of delayed hypersensitivity (DTH) to *Leishmania* antigens (Solano-Gallego et al., 2000), decreased numbers of T cells in the peripheral blood (Martínez-Moreno et al., 1995; De Luna et al., 1999), absence of interleukin 2 (IL-2), and absence of IFN-γ production (Santos-Gomes et al., 2002). The main mechanism involved in the protective immunity of dogs with *L. infantum* is the activation of macrophages by IFN-γ and TNF-α to clear intracellular amastigotes via nitric oxide–arginine pathway. IL-12p40 and IL-2 and IFN-γ are also implicated in delaying disease onset in these animals, IL-12 being detected in lymph node cells from dogs protected against *L. infantum* after immunisation with LACK-expressing vaccine (Ramiro et al., 2003). IL-10 production was correlated with active disease, and IL-2 and IFN-γ were predominantly reported in asymptomatic dogs. IL-17 released by Th17 cells increases IFN-γ production and reduces IL-10 (Rodriguez-Cortes et al., 2017).The active disease is associated with high antibody levels and an escalating Th2 immune response, accompanied by a robust inflammatory reaction (Rodriguez-Cortes et al., 2017) (**Figure 1**). In the clinical phase of the disease, excessive production of IL-17 results in the recruitment of an excessive number of neutrophils to sites of inflammation. This phenomenon causes tissue damage, which is particularly noticeable in the cutaneous and mucocutaneous forms of human leishmaniasis (Toepp and Petersen, 2020). The expression of Toll-like receptors (TLRs) on the surface of monocytes, dendritic cells, and macrophages can trigger either activation or inhibition of cellular functions and the production of IL-12. This, in turn, can lead to a Th1 type response that limits or halts the intracellular survival of parasites, and that can be determined by measuring the proliferation of CD4+ T cells and the production of IFN-γ, which activates macrophages (Strauss-Ayali et al., 2005).

The cytokine IL-4 was not observed in asymptomatic dogs, but was observed in symptomatic dogs, isolated from bone marrow aspirates of dogs which had more severe symptoms (Quinnell et al., 2001). IgG1 and IgG2 subclasses were used more than total IgG as an indicator of canine visceral leishmaniasis status (Deplazes et al., 1995).

High levels of anti-*Leishmania* IgG1 antibodies and the appearance of clinical IgG2 antibodies were detected in symptomatic dogs, as well as IgE (Barbieri, 2006).

CD8+ lymphocytes were observed in asymptomatic dogs that were experimentally infected *with L. infantum*, as well as in immunized dogs, but not in symptomatic dogs. Genetic factors are also considered to play a significant role in the development of the disease, so polymorphisms of the canine NRAMP1 gene (Altet et al., 2002) and MHC class II alleles (Quinnell et al., 2003) have been shown to be important in the development of the disease, so that some breeds of dogs, such as the Ibizan Hound, do not develop signs of symptomatic leishmaniasis, which must be considered when working to control this disease. In canine patients, CD4+ Th1 lymphocytes activate macrophages to effector cells, leading to the production of *Leishmania*-specific IFN-γ. This process allows the host to combat the parasite and manage the advancement of the disease (Toepp and Petersen, 2020). In general, dogs that experience clinical disease and poor clinical outcomes typically exhibit a reduction or absence of the cell-mediated immune response (Solano-Gallego et al., 2016; Martínez-Orellana et al., 2017; Zribi et al., 2017). In “resistant” dogs, the Th1 immune response is dominant and is marked by IFN-γ. However, some of these dogs may not produce antibodies or may have very low levels (sometimes below the cutoff), yet parasite DNA can still be detected in tissues such as bone marrow and lymph nodes in some of these individuals (Oliva et al., 2006; Lombardo et al., 2012).

In order to achieve immunological control of *Leishmania infantum*, that is the main species causing canine leishmaniasis, a state of equilibrium between inflammatory and regulatory responses is necessary. This balance usually occurs between proinflammatory Th1 CD4+ T cells that are responsible for managing parasite multiplication and regulatory T cells 1 which produce immunosuppression, necessary to alleviate exaggerated inflammation, but which if predominates, leads to the progression of canine leishmaniasis. Multiple factors are involved in the development of the disease, for example hunting dogs are more susceptible to developing the disease due to frequent co-infections acquired during hunting and poor constitution. Those in whom the disease does not progress remain in a subclinical state. The presence of parasites in peripheral blood can be identified through qPCR and/or detection of humoral immune responses. Although ELISA may reveal low antibody titres, a robust cellular immune response, including CD4+ T cell proliferation in response to parasite antigen, is observed.

CD4+ T cells have a major role in intracellular control of pathogens by generating IFN-γ-activating macrophages. Th1 cells also contribute to the production of IL-3, CXCL2 and TNF-α, which play an important role in the recruitment, maintenance and differentiation of macrophages at the location of the infection, as well as IL-2 cells, which lead to the formation of more T cells, creating a favourable environment inside cells for the destruction of *Leishmania*. In the course of subclinical disease, canine patients have proliferative cytotoxic CD8+ T cells, which help control *L. infantum* by eliminating infected macrophages (Alexandre-Pires et al., 2010). Studies performed by qPCR showed that even in subclinical form, dogs had increased levels of IL-18 and IL-6 in peripheral blood mononuclear cells, which aided IL-12 in macrophage activation (Solcà et al., 2016). With the progression of leishmaniasis, the immune system is no longer able to maintain a balance in the inflammation process without producing pathology (Boggiatto et al., 2010).

A study carried out in the USA by Boggiatto in 2011, demonstrated that vertical transmission in dogs was the main route of transmission of leishmaniasis, a fact that influences the long-term immunity and clinical presentation of the disease in dogs born from a positive bitch. In 2015 a study showed that a series of dogs from the same litter developed different immune responses, parasites being detected through PCR parasites within two to three years of life and with an interval of six months between each other. The serological patterns were different for each dog, from consistently positive titres to suspicious titers before becoming negative. Negative titres are correlated with maintaining the subclinical state of the disease and have not been correlated with the clinical progression of the disease (Boggiatto et al., 2010; Boggiatto et al., 2011; Solano-Gallego et al., 2011; Vida et al., 2016). Likewise, the assessment of the Th 1 response to parasite antigen proliferation and IFN- production by Fluorescence-Activated Cell Sorting (FACS) analysis, was different with a dramatic increase in serological IFAT, followed by a decrease in T cell proliferation and IFN production; qPCR did not provide informative data on disease progression. Immune responses to vector-borne canine leishmaniasis are generally well known, but very little is known about how vertical transmission modifies responses to infection (Oliveira et al., 2010). The lack of continuous parasite detection by qPCR suggests that the protozoa was intermittently present or intermittently detectable in peripheral blood and evolving with visceralization. Consistent positive serological results suggest antibody-based immune responses to *Leishmania*, but more correlated with clinical progression of the disease than an immune control of the disease (Boggiatto et al., 2011; Toepp et al., 2019a).

As time passes without infection control, canine *L. infantum* -specific CD4+ T cells have been shown to lose antigen proliferation and IFN production, known as T cell exhaustion (Esch et al., 2013). Besides the CD4+ T cells as a source for IFN, CD8+ T cells, TH0 cells and NKT cells can also secrete IFN- during *Leishmania* infection (Esch et al., 2013), however they only have a minor role in the pool - the IFN cytokines. Increased IFN production shows increased proliferation of CD4+ T cells, demonstrating that Th1 immune responses are critical for controlling *Leishmania* and other intracellular pathogens (Sacks and Noben-Trauth, 2002). Dogs infected through vertical transmission may not show the disease after birth and become positive for PCR and serology after 2-3 years, followed by the development into oligosymptomatic after 5 years, these facts emphasizing the need for testing even in the conditions of persistent repellent treatments against phlebotome stings (Gavgani et al., 2002).

The clinical manifestations described in leishmaniasis are dependent not only on the genetic background of the host, on its immunocompetence and nutritional status, but can also be influenced by the parasite species that initiates the infection, as well as by the vector and some social factors (Blackwell et al., 2020).

## 5 Diagnosis in canine leishmaniasis

The diagnosis of canine leishmaniasis is intricate and multiple strategies are required to be used. In endemic zones, it is recommended to employ the clinical diagnosis in combination with specific techniques according to the clinical signs and laboratory results, such as quantitative serology (IFAT or ELISA). This is to examine the humoral response, because in clinical leishmaniasis the antibody levels are elevated. In addition, along with parasitological diagnosis, molecular diagnosis is used to detect specific DNA through biopsy and/or cytology (conventional PCR, qPCR, nested PCR) (Solano-Gallego et al., 2016).

As in human leishmaniasis, in CVL diagnosis, the gold standard remains the parasitological diagnosis that includes the microscopic examination of smears and aspirates from the spleen, liver, bone marrow, lymphatic nodes, and of biopsy material from damaged or intact skin (Pinelli et al., 1994; Barbieri, 2006). But this method also has a number of drawbacks, the result depending on the experience of the observer, the parasite load, as well as the immune response developed by the host (Solano-Gallego et al., 2000). The most used are serological tests, but this tests present a series of disadvantages, especially those based on parasitic antigens, as they cross-react with other *Leishmania* species, and also with *Trypanosoma* species (De Luna et al., 1999; Barbieri, 2006). Several recombinant proteins, including rLb6H from *Leishmania braziliensis*, have been tested and found to be effective in diagnosing human leishmaniasis. The use of this protein has produced positive outcomes in diagnosing visceral leishmaniasis caused by *Leishmania infantum* and American cutaneous leishmaniasis (ACL) resulting from infection with species of *Leishmania* such as *L. braziliensis, L. amazonensis*, *L. shawi*, and *L. guyanensis*. However, these proteins still have drawbacks due to their tendency to produce cross-reactivity with sera from patients with toxoplasmosis, malaria, Chagas disease, paracoccidioidomycosis, tuberculosis, and histoplasmosis (Martínez-Moreno et al., 1995). rKLO8, a kinesin protein derived from *Leishmania donovani* found in Sudan, has demonstrated strong specificity and sensitivity, particularly in patients with VL in India and East Africa (Pinelli et al., 1995). Furthermore, the utilization of both rKLO8 and rK26 proteins in combination has enhanced the specificity and sensitivity of serodiagnosis for CVL, surpassing that of monospecific ELISA (Santos-Gomes et al., 2002). The most used are the fusion proteins rK39 and rK28, also used successfully in human medicine; rK28 (obtained from the fusion of rK9, rK26 and rK39 from *L. donovani*), being recommended by the Brazilian Ministry of Health since 2011, in the form of a rapid immunochromatographic test. In the diagnosis of leishmaniasis in dogs, it is extremely important to differentiate vaccinated dogs from those infected with *Leishmania.*

Dogs who presented a series of clinical signs came out positive in the ELISA and IFAT tests, making these tests reliable in the diagnosis of clinical leishmaniasis (Priolo et al., 2022).

The use of the Real Time PCR technique for the detection of *L. infantum* proved clearly superior to the conventional PCR technique, which failed to detect *Leishmania* DNA in samples with a drecreased load of parasites (Mary et al., 2004; Rolão et al., 2004). Consequently, studies utilizing polymerase chain reaction (PCR) to detect DNA in different canine tissues have reported increased rates of positivity (Priolo et al., 2022). A study carried out by Vito Priolo in 2022 (Priolo et al., 2022) shows that there are no differences according to PCR positivity among a series of studied variables such as the age, gender and race of the patients taken into account. Nested and semi-nested PCR techniques are crucial for distinguishing between different species. This method involves the use of two sets of primers in two consecutive cycles, wherein the second set amplifies the secondary target in the first product. Despite being highly sensitive, this approach has limitations due to the possibility of contamination (Akhoundi et al., 2017).

## 6 Discussion

Immunoassays were the most personalised, being appropriate for endemic regions, but still unable to distinguish active infections. In addition, molecular methods have high specificity and sensitivity but do not indicate whether the infection is active. The parasitological techniques, which are considered the gold standard in the diagnosis of leishmaniasis, have a number of drawbacks as they require time, qualified personnel, and in the case of an asymptomatic infection where the parasite load is low, the identification is limited, with the risk of a false negative response. In asymptomatic cases, antibody titres are also low, and serological tests are limited as in HIV co-infections. Therefore, the diagnosis and surveillance of leishmaniasis cases remains an open topic, with the need for new methods adapted to the immunological status of the host.

In countries where sandfly species exist, it is believed that both humans and dogs who have a subclinical form of leishmaniasis play a significant role in spreading and transmitting the disease (Nylén and Sacks, 2007). In order to reduce the progression and transmission of the disease, it is very important to maintain the functionality of the T cells, trying through vaccines and specific therapies to eliminate the disease. Chronic exposure to *Leishmania* protozoan antigen can lead to T-cell exhaustion, which can be reactivated by using a vaccine antigen and TLR agonists.

Recent studies have proposed the potential use of vaccines in combination with chemotherapy as immunotherapies for infected dogs. There are 3 canine leishmaniasis vaccines available and used on the market, namely: CaniLeish^®^ (Virbac Santé Animale, France), Leish-Tec^®^ (Ceva Animal Health, Brazil), and Letifend^®^ (Laboratorios Leti, Spain) (Solano-Gallego et al., 2017), with the specification that the manufacturers only recommend vaccinating seronegative dogs. The challenge is the difficulty of identifying completely healthy dogs due to the existence of gaps in current diagnosis. A study conducted by Toepp et al. in 2018 (Toepp et al., 2018) showed that adverse effects were low, approximately 3%, when vaccinating healthy subclinical dogs with LeishTec, a study which should be considered and used in the vaccination/immunotherapy protocol of infected healthy animals. But it must be taken into account that during the sandfly season, other vectors such as mosquitoes and ticks are also active, which can transmit a number of pathogens such as *Dirofilaria* spp., *Babesia* spp., *Ehrilichia* spp., *Anaplasma* spp.; all these pathogens can evolve as co-infections with *Leishmania*, that affects the immune system and can lead to complications of leishmaniasis (Toepp et al., 2019b).

## 7 Conclusions

CVL remains a challenge, as the balance of the immune system is very delicate in trying to prevent the replication and growth of *Leishmania* parasites. Thus, in the attempt to establish a disease control protocol, combinations of vaccination and immunotherapy should also be taken into account, as well as diagnostic protocols, since no diagnostic method has proven to be 100% safe in asymptomatic CVL. Consequently, for the diagnosis of the disease in endemic areas, combinations of diagnostic methods should be established, adapted to the region where the disease evolves.

This problem is also encountered in human medicine, especially if we talk about patients with HIV, or other immunosuppressive diseases, which affect the immune system differently in VL.

In conclusion, both human and canine leishmaniasis remains a disease which requires detailed research regarding the response of the immune system in various conditions, as well as the establishment of safe diagnostic methods.

Consequently, it is necessary to introduce this disease to the list of diseases of great interest also in the context of global warming and the movement of the human and animal population, which increase the risk of global dissemination.

## Author contributions

LI: Conceptualization, Data curation, Investigation, Methodology, Supervision, Writing – original draft. BA: Conceptualization, Funding acquisition, Investigation, Writing – original draft. SG-H: Funding acquisition, Investigation, Resources, Supervision, Writing – review & editing. GM: Funding acquisition, Methodology, Resources, Writing – review & editing. RM: Funding acquisition, Investigation, Resources, Visualization, Writing – review & editing. LM: Conceptualization, Data curation, Project administration, Supervision, Writing – review & editing.
